# Time Preferences and Natural Resource Extraction Behavior: An Experimental Study from Artisanal Fisheries in Zanzibar

**DOI:** 10.1371/journal.pone.0168898

**Published:** 2016-12-29

**Authors:** Aneeque Javaid, Micaela M. Kulesz, Achim Schlüter, Alexandra Ghosh, Narriman S. Jiddawi

**Affiliations:** 1 Institut für Umweltsystemforschung, Universität Osnabrück, Osnabrück, Germany; 2 Jacobs University, Bremen, Germany; 3 Leibniz center for Tropical Marine Ecology (Zmt), Bremen, Germany; 4 Swedish University of Agricultural Sciences, Uppsala, Sweden; 5 Institute of Marine Science, Dar-es-Salaam University, Zanzibar, Tanzania; Middlesex University, UNITED KINGDOM

## Abstract

Natural resource users face a trade-off between present and future consumption. Using harmful methods or extracting unsustainably, lowers future consumption. Therefore, it is reasonable to posit that people with higher time preferences extract more as compared to people with lower time preferences. The present study combines experimental methods and questionnaire data in order to understand the relationship between individual time preferences and fishers’ extraction behavior. We elicit individual time preferences using an incentivized experiment, linking the resulting time preference measures to extraction data from a questionnaire, as well as data collected from a framed Common Pool Resource (CPR) experiment. Both the experiments and questionnaire were conducted with artisanal fishers in Zanzibar. Our findings suggest that the relationship between time preferences and CPR extraction is not as straightforward as predicted by classical economic theory. In contrast to earlier studies, we find that fishers’ time preferences are negatively correlated to their extraction rates. Our surprising findings can partly be explained by the fact that higher time preferences are associated with lower investment in extraction capability (the disinvestment effect of time preferences), and by fishers´ cognitive abilities.

## Introduction

Classical economic theory suggests that resource extraction is an inter-temporal maximization problem, where time preferences (i.e., the preference for immediate utility over delayed utility) are crucial in determining the overall extraction rate [1]. Individuals weigh their utility derived from extracting the resource in the current time period against the utility derived from conserving the resource for future consumption. In this way, time preferences enter an individual´s decision-making process in the form of an individual discount function (this includes both the shape of discount function as well the discount factor). Over-extraction in the current time period reduces the future availability of the resource. It is generally accepted that lower discount factors accelerate extraction by decreasing the value given to the future resource condition [2, 3]. This paper tests whether individual time preferences correlate with extraction behavior in a natural resource setting. In particular, we investigate whether lower discount factors and the presence of present bias are related to higher extraction behavior among natural resource users.

Earlier empirical research demonstrates that there exists significant variation amongst resource users with regards to their time preferences [4–7], as well as their resource extraction behavior [8–11]. Fehr and Leibbrandt's [12] seminal work examining the relationship between time preference measures and resource extraction shows that resource users who exhibit impatient behavior are more likely to accelerate the process of resource depletion. In a similar vein, Johnson and Saunders [6] find that time preference measures are able to predict resource management preferences. These empirical studies, which employ experimental methods, provide evidence that impatient and myopic individuals are more likely to engage in unsustainable resource extraction.

In spite of these compelling findings, earlier empirical research is limited in two ways. First, most empirical studies use non-incentivized or primary rewards-based tasks that do not account for the degree of heterogeneity in time preferences (e.g., Fehr and Leibbrandt [12]). Second, both the existing theoretical and empirical literature focus on natural resources with private property rights. For example, Fehr and Leibbrandt [12] look at the relationship between time preferences and resource extraction in the case of a (weak) private property situation. However, most natural resources, especially in developing countries, are not managed as a private property. Rather, they are managed as what could be termed as a common property regime (CPR)[13]. While the theoretical predictions about the impact of time preferences on extraction behavior are quite clear in the case of private property rights, they are more ambiguous in the case of common-pool resources. This raises the question as to whether or not earlier findings about time preferences and resource extraction are applicable to common-pool resources as well.

Following this line of research, the present study examines the relationship between natural resource users’ time preferences and their relative extraction rates. We investigate the case of a fishery resource, which is evidently a CPR system of major global importance. This study contributes to earlier literature by focusing on the case of a common pool resource situation, and also by using a more realistic measure of time preferences based on multiple price lists (MPLs). Furthermore, we combine questionnaire and experimental data, which, taken together, increases the robustness of our findings.

Our results suggest that the relationship between time preferences and natural resource extraction is not as straightforward as assumed by classical economic theory. We find that time preferences are negatively correlated to extraction rates in the CPR experiment. Our findings are partly explained by the disinvestment effect of time preferences as well as the variation in the respective cognitive abilities of fishers.

## Research Design

**Fig 1:**
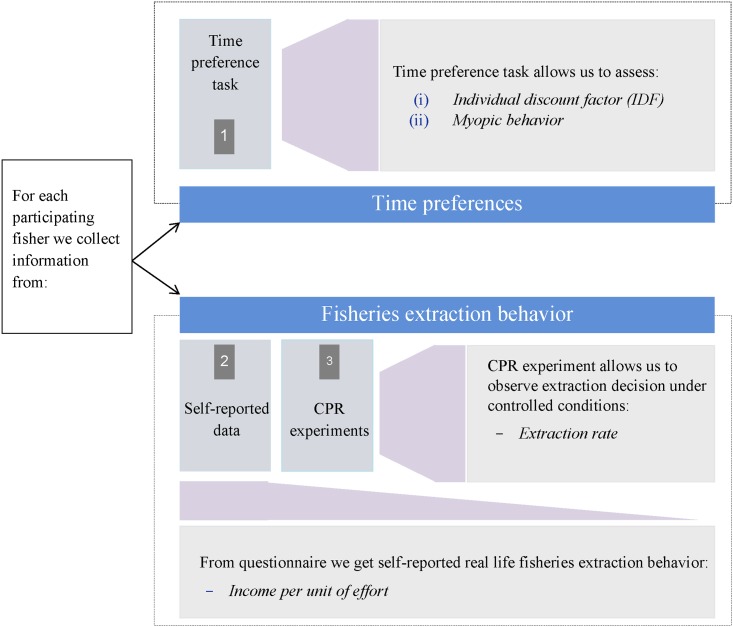
Summary of research design.

Based on earlier theoretical and empirical work (see Introduction), we guide our study on the hypothesis that fishers exhibiting higher time preferences are more likely to engage in activities that result in greater extraction from the natural resource. It should be noted that while earlier theoretical and empirical studies explicitly examine this relationship in the context of private property regimes, there is a case to be made that these findings are relevant for common property regimes as well. For instance, we know from earlier literature that cooperation is fundamental for sustainable common pool resource management [14, 15]. Axelrod [16] hypothesized that in order to facilitate cooperation, particularly in repeated social dilemma situations, patience is a key characteristic, as patient individuals are more likely to forgo the immediate benefits of selfish behavior and look at the greater future benefits associated with cooperation. Building on this hypothesis, Al-Ubaydli and Jones [17] show that higher time preferences are indeed linked with greater cooperation in stag-hunt games. Based on these observations we hypothesize that the relationship between time preferences and resource extraction in a common property regime is similar to the one observed and hypothesized in private property regimes. Our research strategy is illustrated in Fig 1.

The research design consists of two major components. First, we measured individual time preferences using incentivized multiple price lists, hereafter referred to as the time preference task [4, 18]. Second, we collected data on individuals´ real-life extraction behavior, as well as their extraction behavior under controlled experimental settings.

We conducted our study in 10 separate villages across 5 different fishing districts in Zanzibar. We aimed to select villages with the most important fish landing sites for fishers, and while this means that some districts have greater representation in our sample, their representation reflects the total number of fishers operating in the area. We generally conducted one experimental session per village, although, in the case of big villages, we divided the village into two parts and conducted one experimental session in each part.

We collected data for a total of 240 fishers, however, approximately 15–20% of participants made inconsistent choices in the time preference task and, as a result, were not considered in our empirical examination. Therefore, for our main analysis, we focus on the remaining 188 fishers. All of the participants were fishers recruited from local fishing markets. Almost 95% of the participants depend on fishing activities as their primary source of income. The average age in our sample is 41 (±15) years, and average fishing experience is 21 (±14) years. For further details on the composition of our sample, kindly refer to Table B in S1 File.

During each experimental session, we first conducted the time preference task, which was then followed by the common pool resource (CPR) experiment. At the end of each session, participants took part in an incentivized risk preference task [19]. The experimental sessions, including the questionnaire, lasted between 2 to 3 hours. The payments from the CPR experiment and the time preference task were awarded through cellphone credit. All of the participants in our sample, and the vast majority of cellphone users in Zanzibar operate their phone credit on the basis of a pay-as-you-go model, which means the value of an additional payment is much higher than in the case of fixed contracts. Furthermore, cellphone credit is transferable and can be used to pay for things such as groceries and transportation. Our experiments were designed to make sure that both the average cash earnings and the average phone credit earnings amounted to at least the average income from a full day’s work (approximately 8000–10,000 TZS ~ 5–6 USD).

We used the standard protocols for both the time preference task and the CPR experiment (short experimental protocols are given in the S2 File, while detailed protocols are available upon request). These experiments are pen and paper based experiments, which were conducted by local field assistants under the supervision of the first author. In the case of illiterate participants, the experimental protocols were explained by one of the field assistants. Additionally, an oral quiz was conducted before the experiment to make sure that all of the participants understood the experiment.

Before commencing an experimental session, participants provided their contact information. Furthermore, at this stage, participants gave oral consent to participate in the research activities, and to receive their experimental earnings in the form of cellphone credit. Similarly, at the end of each experimental session, participants provided written consent acknowledging that they had willingly participated in the experiments and the questionnaires, and that they had no qualms with the collection and usage of data for research purposes. Furthermore, participants also signed a receipt indicating their earnings from the experiments, which included both the amount delivered to them immediately after the experiment, and the amount which was to be delivered to them on a later date. This receipt bore the contact information of the experimenters, who could be reached regarding the payments.

We collected identifying information from the participants. This information was only used for delivering participants´ earnings. Additionally, it allowed us to contact participants and verify that they had received their earnings. This data was collected and stored by the first author himself. No other person had access to this data, which was destroyed after all payments had been delivered (approximately 2–3 weeks after the date of the experimental session). All data from this study were then anonymized by the first author.

### Time-preferences

We use an incentivized multiple price list (MPL) task to measure individual time preferences (for similar approaches see Tanaka, Camerer [4]). Time preference measures obtained from MPLs have been shown to (i) be stable at the individual level over time [20–23], (ii) correlate with measures derived from other methodologies used to investigate time preferences [22, 24, 25], and (iii), induce the same neurological responses as primary reward-based tasks [26–28]. Furthermore, they are a better predictor of life outcomes than non-incentivized measures [24, 29]. And finally, MPLs avoid the risk of temptation-based negative aspects of primary reward-based tasks [25]. However, it must be acknowledged that MPLS are not without limitations. Recently, some critics have pointed out the potential problems of using this method and proposed other methods to address these issues (see for example Andreoni and Sprenger [30]). However, during our initial pre-testing we found that MPLs were easier to understand for participants than other comparable methods of measuring time preferences. For this reason we believe that MPLs, while far from being ideal, provide a somewhat reliable estimation of participants’ time preferences.

In our time preference task, subjects are required to make 10 choices between smaller rewards (X) delivered at time t1 (option A), and larger rewards (Y) delivered at time t2 (option B), where t2 is always greater than t1. Choices are grouped into two lists: (1) t1 = day 0 and t2 = day 14; and (2) t1 = day 1 and t2 = day 15. For each choice, Y remains constant while X varies from smaller to larger amounts. At the beginning of each session, participants were informed that only one out of the 10 choices would be randomly selected for payment.

The MPL task allows us to account for two different dimensions of individual time preferences: (1) *patience* (*impatience)*, which is captured by the individual discount factor (IDF), and (2) myopic behavior, which indicates that an individual is present-biased. We calculate these two measures as follows. For each list, we estimate the individual discount factors (IDFs) based on the choice at which an individual switches from opting for the smaller, more immediate payment to the larger, later payment. In the extreme cases, if a participant always chooses option X in the list, we designate her IDF_t1,t2_ to be 0.125. On the other hand, if a participant always chosen option Y, we designate her IDF_t1,t2_ to be 1. This procedure results in two distinct discount measures, IDF_0,14_ and IDF_1,15_. We use the average of these as the first time preference measure, hereafter “IDF”. Having two lists allows us to identify time inconsistency in the form of myopic behavior, and to distinguish present-biased participants: an individual is classified as present-biased when her IDF_0,14_ < IDF_1,15_.

The average IDF for fishers in our sample is 0.63, and approximately 20 percent of our participants can be classified as present-biased (Table 1). These estimates are comparable to the ones found in earlier studies investigating the time preferences of fishers [5, 6].

**Table 1:** Summary of Time Preferences and Extraction Behavior.

	Variable	Average	Std. dev.	%	Min	Max
**Time Preferences**						
	*IDF*	0.63	0.36	-	0.125	1
	*Present-biased*	-	-	20%	0	1
**Extraction Behavior**						
Self-reported data:	*income per unit of effort (Tsh*.*/hours)*	3496.34	3047.21	-	286	16667
CPR experiment:	extraction rate (tokens per round)*	75	50	-	0	160

* The experiment was run with tokens. At the end, participants received 10 Tanzanian Shillings (TZS) per token earned.

### Extraction data

#### Self-reported

We collected individual extraction data with the use of extensive questionnaires. We asked fisher questions regarding their average income as well as their average effort levels (hours per day) for the normal, slow, and high seasons. The extraction behavior was calculated based on the mean value of these income and effort variables. The questionnaire also includes socio-economic and demographic information (see Table A in S1 File).

We choose to use the income measurement as it was considered to be the most reliable and standardized way of eliciting the information regarding extraction behavior. Not only was this advised by local fisheries experts with whom we discussed at length, but also fishers´ marketing and selling abilities play a negligible role in determining income at the local level. Most of the fishers in our sample leave this responsibility to others, only observing the sale of their catch. The variation in income from fishing activities is mostly a result of using higher intensity gears and the ability to use these gears in a more extractive way. Extraction rate is thus captured by income per unit of effort or productivity. This is an indicator for (1) fishers´ intensity of effort, and (2) fishing skills, which encompass proper maintenance and operation of gears and boats. Table 1 provides the general descriptive statistics.

#### CPR experiment

We conducted a CPR experiment based on Cox andOstrom [31]. The experiment was framed as an extraction activity from a common fishery. Participants took part in the experiments in groups of 6, where group members were known to each other. The CPR game was repeated for 5 rounds. In each round individuals simultaneously and privately decided on how many tokens to extract from the common resource.

Player earnings are given as:

$$\pi_{i}=\left(X_{i}\right)+Y$$

where $Y=\left(R-\sum_{i=1}^{n}x_{i}\right)/n$

X_i_ are the earnings from resource extraction which are equal to the individual extraction level (x_i_), Y are the earnings from conservation, R is the amount of the common resource, and *n* is the number of individuals sharing the resource.

Extraction from a natural resource involves a trade-off between present and future consumption. Specifically, the benefits of conservation are delivered in the future, while the benefits of extraction are available immediately. In a standard CPR experiment, this aspect is neglected since participants are paid their extraction and conservation earnings at the end of the experiment and do not have to wait for the conservation benefits, unlike real life situations. Our design accounts for this time lag by including a treatment, hereafter referred to as the “Time treatment”, where earnings from conserved resource (Y) are delivered 14 days after the end of the experimental session. Earnings from private extraction (X_i_), however, are delivered immediately at the end of the experimental session. In order to ensure that the conditions were as equal as possible between the standard and the time treatment groups, we paid both groups the conservation benefit via telephone credit. Table 2 illustrates the CPR experimental design. Note that groups were randomly assigned to the time treatment or to the control group. In Table K in S1 File, we show that there are no significant differences in fishermen characteristics between the treatment and the control groups.

**Table 2:** Experimental Design.

	Control Groups	Treatment Groups
Time Treatment	No	Yes
# of groups	20	20
# of participants	120	120
# of rounds	5	5

The mean extraction rate per round of the CPR experiment is 75 tokens, equivalent to TZS 750 (for reference, 1 USD ~ 1650 TZS), which is approximately 45% of the maximum extraction rate. For more details on extraction behavior, kindly refer to Table 1.

## Results

### Fisheries extraction and time preferences

To test our hypothesis that fishers´ time preferences are positively correlated with their extraction behavior, we begin by looking at the self-reported extraction data. We carried out the study focusing on fishers’ productivity, indicated by the income per unit of effort (IUF), where one unit of effort corresponds with one hour of fishing activities. We estimate using Ordinary Least-Squares (OLS) regression models, where *IUF* is the dependent variable, and *IDF* (representing the degree of patience) and *Present-biased* (representing the presence of myopic behavior) are the main independent variables of interest. Table 3 reports the results.

**Table 3:** Regression models for fisheries income.

	*Ln (income per unit of effort)*					
	(1)	(2)	(3)	(4)	(5)	(6)
IDF	0.103 (0.190)	-	0.118 (0.190)	0.0790 (0.189)	-0.0287 (0.186)	0.0220 (0.203)
Present biased (= 1)	-	-0.287* **(0.146)**	-0.292** **(0.147)**	-0.273* **(0.151)**	-0.295* **(0.154)**	-0.449*** **(0.157)**
Risk averse (= 1)	-	-	-	-0.286* **(0.156)**	-0.320** **(0.154)**	-0.172 (0.151)
Cons.	7.905*** **(0.125)**	8.030*** **(0.0817)**	7.957*** **(0.128)**	8.173*** **(0.174)**	8.019*** **(0.395)**	8.204*** **(0.465)**
Sociodemographic indicators	No	No	No	No	Yes	Yes
Fisheries related variables	No	No	No	No	No	Yes
R2	0.001	0.014	0.016	0.034	0.094	0.183
N	188	188	188	188	188	185

*Notes*: (1) OLS regression model where dependent variable is income per unit of effort from fishing activities.

(2) Robust standard errors in parentheses.

(3) * *p* < 0.1,

** *p* < 0.05,

*** *p* < 0.01.

#### Result 1: Myopic fishers exhibit lower extraction rates

We run six different specifications of our OLS model. In Columns (1) and (2), we present the results of regression models whereby IDF and present-bias are the independent variables in isolation, respectively. In Column (3), we include both IDF and present-bias, and in Column (4) we control for attitudes towards risk. Finally, in Columns (5) and (6), we noticeably increase the explanatory power of our model by controlling for sociodemographic characteristics and fisheries related variables, respectively.

Classical economic theory would predict that more impatient and myopic fishers are not concerned about the future state of the resource and are thus more likely to engage in higher levels of extraction than their counterparts (cf. Introduction). Contrary to the theoretical predictions, however, we find that impatient and myopic fishers exhibit lower extraction rates, as indicated by (i) the positive coefficient for IDF and (ii) the negative coefficient for present bias. However, when looking at significances and magnitudes, only present-bias drives our findings, while IDF remains insignificant. This suggests that fishers’ valuation of future consumption does not necessarily define their extraction from the common resource.

The relationship between time preferences and extraction rates can be affected by attitudes towards risk [30, 32]. Using Maier and Rüger [19]’s multiple price method we identify fishers who are risk-averse and those who are not. When controlling for risk preferences (Table 3, Column (4)), we observe that the coefficient for risk is negative, which indicates that risk-averse fishers are less productive than their non-risk-averse peers, however this does not change our result regarding the effect of present bias on extraction rates. Moreover, Result 1 is robust when controlling for sociodemographic indicators and fisheries-related variables such as age, education level, boat type, etc. (see Table C in S1 File for further details).

### CPR experiment and time preferences

Previous research emphasizes the difficulties associated with self-reported data when it comes to analyzing fishers’ extraction behavior. Indeed, illegal and under-reported fishing is one of the biggest challenges facing fisheries management [33, 34]. According to recent estimates, actual extraction rates in some regions of Africa are 30–50% higher than the officially reported catch rates [34–36]. In order to overcome the limitations associated with this methodological approach, we implement a CPR experiment where participants make extraction decisions in a controlled setting using tangible, monetary incentives.

#### Result 2: Impatient fishers exhibit significantly lower extraction rates

We test our hypothesis using fishers’ extraction rates collected from the CPR experiment. We estimate using a random-effects model where extraction per round is the dependent variable. Table 4 presents the results. However, as a robustness check we estimate this relationship using a pooled OLS regression model as well (Table E in S1 File), where we assume that fishers’ preferences remain constant over the five rounds of our experiments. Nevertheless, our findings remain consistent in both models. As before, full models with sociodemographic indicators and fisheries-related variables are shown in Table D in S1 File.

**Table 4:** Random-effects regression model for damage done to the CPR.

	*Extraction rate*					
	Control groups			Time Treatment groups		
	(1)	(2)	(3)	(4)	(5)	(6)
IDF	0.138 (10.81)	-	0.777 (10.51)	23.78** (11.07)	**-**	29.81*** (9.005)
Round number	2.468** (1.258)	2.468** (1.258)	2.468* (1.262)	0.957 (1.439)	0.957 (1.439)	0.957 (1.443)
Present biased (= 1)	-	-7.684 (13.12)	-5.118 (13.12)	-	3.652 (9.907)	1.800 (10.31)
Risk averse (= 1)	-	-	-2.795 (7.959)	-	-	-12.82* (6.967)
cellphone (= 1)	-	-	-20.6*** (5.107)	-	-	-19.98* (10.72)
Cons.	67.51*** (7.664)	68.99*** (5.900)	86.07*** (9.114)	67.21*** (11.79)	81.29*** (10.12)	87.87*** (13.41)
*R*^2^	0.04	0.06	0.04	0.03	0.01	0.08
No. of players	94	94	94	94	94	94
No. of obs.	470	470	470	470	470	470

*Notes*: (1) Random effects panel regression model where dependent variable is extraction rate per round.

(2) Cluster robust standard errors in parentheses

(3) * *p* < 0.1, ** *p* < 0.05, *** *p* < 0.01.

(4) Column 1–3 looks at the control groups only whereas column 4–6 look at the Time Treatment groups.

Table 4a and 4b report the results for the control and the treatment group, respectively. Note again that the treatment groups received their conservation earnings after a 14-day delay (cf. Research design).

For the Time treatment groups (Table 4b), we find that the coefficient for IDF is positive and statistically significant (at the 5% level), indicating that on average, relatively more patient fishers exhibit higher extraction rates. The coefficient of IDF is 20% of the maximum possible amount that can be extracted per round, which suggests a large difference in extraction rates between patient and impatient fishers.

For the control group we find no evidence of extraction rates being related to fishers’ IDF or to their present-bias. This finding is to be expected, as standard CPR experiments do not include heterogeneous time preferences in their designs, and thus do not provide realistic incentives to study the effect of time preferences on extraction rates. Indeed, the motivation to include the time treatment was to address this limitation. Even though our results are not aligned with the theoretical prediction, they prove to be consistent with each other: both self-reported data and revealed behavior in the experimental setting show that having a higher patience level is not associated with greater resource conservation, rather, in both cases, relatively impatient and myopic individuals extract lower amounts than their counterparts.

In order to make sure that our results are robust to different modeling choices, we perform additional robustness checks. These robustness checks include (i) calculating models with clustered standard errors at the village and district level, (ii) including district dummies and variables indicating village and district level characteristics, (iii) including participants with inconsistent time preferences by calculating their discount rate based on the first switching point in each list, and (iv) including group level information about the previous rounds of extraction in the CPR experiment. These models are presented in S1 File under the section “robustness checks” (Tables H-J in S1 File). Overall, we find that our results are robust to these different variations, and qualitatively, the relationship between time preferences and extraction behavior remain the same regardless of the choice of the model.

## Discussion

A recent strand of literature investigating the effect of time preferences on natural resource extraction behavior shows that impatient individuals engage in more intensive resource extraction activities. Our findings suggest otherwise; patient fishers extract more than impatient ones. In this section, we provide potential explanations for this result, which follows with a discussion on the limitations of our study.

### Disinvestment effect

Farzin [37] argues that time preferences can impact extraction behavior in two distinct and countervailing ways: (i) the conservation effect, where high time preferences (i.e., impatience) render future consumption substantially less attractive, and (ii) the disinvestment effect, where high time preferences lead to lower investment in extraction technology. According to Farzin [37] the disinvestment effect could be in terms of: (i) investment in developing a substitute and, (ii) investment in building capacity to extract more resources. Since all of our sample consists of people with fishing as their primary, and in most cases their only major source of income, we focus on the disinvestment effect only in terms of investment in building capacity to extract resources. Indeed, building up the capacity to extract large amounts of fish requires a substantial investment in buying and maintaining fishing equipment. A rational fisher discounts the future value of her investments and then chooses how to allocate her resources between present consumption and investment for the future. Taking this into consideration, an impatient fisher will overweigh the value of her benefits in the present, finding present consumption hard to resist. In every time period, an impatient fisher will prefer to consume today, and thus enjoy her benefits today, instead of investing in fishing gears and collecting the benefits in later periods. Fisheries in Zanzibar are generally characterized as small-scale and artisanal. Most fishers do not count on large amounts of disposable income, which has a negative impact on their ability to increase their fishing capabilities. Based on the promise of higher future income, relatively patient fishers are more willing to accept reduced current consumption and invest in fishing capabilities more than relatively impatient ones. Given that the difference in productivity between patient and impatient fishers stems from using high impact and costly gears, the disinvestment effect dominates the conservation effect in small-scale artisanal fishery settings such as the one under study. In order to test this hypothesis we looked at the investments made by fishers. For the purpose of this study, we focused solely on the investment in fishing gears as this is one of the most important factors in determining a fisher´s extraction level. However, obtaining reliable information about investment in fishing gear proved to be a difficult endeavor. Only a limited number of people in our study (160 out of total 240 fishers) could provide reliable cost estimates about their investment in fishing gears. Regression analysis based on this sub-sample confirm that in accordance with the disinvestment effect, higher IDFs are associated with higher investment in fishing equipment (Table G in S1 File).

### Cognitive abilities

Previous studies argue that intelligence level is linked to patience [17] as well as cooperative behavior in Prisoner’s dilemma or Public goods games [38]. We explore further along these lines by looking at the relationship between extraction level and cognitive ability. Given the high correlation between general intelligence and working memory [39, 40], we ran an incentivized memory task as a proxy for cognitive ability and then regressed it against fishers’ extraction rates, as well as fishers’ time preferences. Note that this measure of cognitive ability is different and independent from participants’ comprehension of experiments, which was assessed using questions related to experiment protocols. Our results (Table F in S1 File) show that the average performance in the memory task is positively associated with both the IDF as well as the extraction rate in the CPR experiment. In other words, fishers exhibiting higher cognitive ability are more patient and extract more. We argue that patient fishers, who also have higher cognitive ability, view natural resource extraction from a completely different perspective than impatient fishers with lower cognitive ability. Fishers with higher cognitive abilities are better able to predict the actions of other fishers, and therefore adapt their own behavior in order to pursue their desired outcome, particularly in an experimental setting [41]. They exploit fisheries in order to smooth their consumption over time and to do so they invest in fishing capabilities, which allows them to extract more in the future. This investment provides greater flexibility and opportunity, thereby reducing the risk of non-extraction in the future, for instance, in the event of an exponential increase in the fisher population. In contrast, fishers with lower cognitive abilities do not plan for the future, and instead apply a subsistence-based heuristic, which leads to lower extraction levels.

It should be noted that the main purpose of our study is to establish a correlation between time preferences and natural resource extraction. So, while our initial hypotheses are based on theoretical considerations (see Research Design) and the available empirical evidence, we argue that individual cognitive ability and time preferences drive the level of investment in extraction capability, and the resulting extraction levels. However, it should be noted that in both cases we cannot rule out important feedback loops. It is possible that accumulating experience with certain gears could lead to lower time preferences. For instance, a fisher working with traditional *dema* baskets may grow accustomed to a longer time horizon in his or her thinking and general planning. However, this is an unlikely scenario as most of the fishers in our sample have experience working with multiple gears, and the likelihood of extraction methods dictating individual preference seems less very low. Similarly, it is possible that a general lack of investment capacity could lead to higher impatience. However, the composition of our sample is quite homogenous in terms of wealth levels, with most of the fishers engaging in subsistence level consumption. Given this, it is unlikely that a general lack of investment capacity could have led to such relatively large differences in time preferences for the individuals in our study.

Therefore, it is reasonable that cognitive ability, in conjunction with the disinvestment effect, provide an explanation as to why patient fishers are less inclined to engage in resource conservation. In this way, our consideration of cognitive ability adds a more nuanced perspective to the discussion on time preferences and resource extraction.

### Limitations and future research suggestions

Our research expands on the relationship between time preferences and resource extraction behavior, yet it has several limitations. First, although we attempted to test the disinvestment effect by assessing fishers’ costs, only a portion of our sample could provide somewhat reliable estimates. Nevertheless, regression analysis based on this sub-sample revealed that, in accordance with the disinvestment effect, patience was associated with higher investment in fishing equipment (see Table G in S1 File). Therefore, we suggest future studies investigate this issue more closely.

Second, we cannot be certain how the conservation and disinvestment effects interact in the long run (i.e., more than 14 days). Since the benefits were delayed for such a short period of time in our experiments, individuals may have given greater consideration to their effort and investment decisions. As resource conservation is generally regarded as a long-term issue, a longer time horizon may lead to greater environmental consideration in the decision-making process.

Third, and finally, our research design does not allow us to completely rule out the possibility that cognitive ability is the main driver behind higher extraction rates. Although we measure cognitive ability using a memory performance task, it does not capture the multi-faceted nature of general intelligence. In addition, in order to disentangle the effects of cognitive ability and patience, future studies should carefully manipulate experimental design to gauge a variety of resource extraction settings.

## Concluding Remarks

Our study sheds light on the importance of the disinvestment effect of time preferences and the cognitive ability of resource users, both of which play a key role in determining the effect that time preferences have on resource extraction behavior, especially under common-pool resource settings. While our results do not come to the same conclusion as earlier studies, they complement and expand on earlier research by emphasizing the significance of the above-mentioned factors. Future research should take into consideration the questions raised by our study, as our findings demonstrate that the relationship between individual time preferences and resource extraction behavior is a complex one, where contextual characteristics can be highly influential.

## Supporting Information

S1 File Detailed regression models. (RTF)

S2 File Short experimental protocols. (DOCX)

S3 File Questionnaire in Swahili. (DOCX)

S4 File Questionnaire in English. (DOCX)

S5 File Data from experiments. (ZIP)
