# Supplementary material for: Time Preferences and Natural Resource Extraction Behavior: An Experimental Study from Artisanal Fisheries in Zanzibar
Source: PLoS One. 2016 Dec 29;11(12):e0168898. doi: 10.1371/journal.pone.0168898 (PMC5199085; doi:10.1371/journal.pone.0168898)
Supplement: S1 File — (RTF) [file pone.0168898.s001.rtf]

Appendix 
Table A: Variable description

IDF.	0-1	An individual´s discount factor which he/she uses to evaluate present value of future outcomes	
Present biased (=1)	0 or 1	Whether or not the individual is dynamically inconsistent	
Risk averse (=1)	0 or 1	Whether or not the individual is risk averse	
Untrustworthy (=1)	0 or 1	Whether or not the individual trust others; thinks are trustworthy or not	
Cellphone (=1)	0 or 1	Whether or not the individual owns a cellphone	
Age		Number of years	
HH members		Number of people living in the same household as yours	
Alternate livelihood		0 = no other livelihood; 1 = farming; 2 = skilled labor
3 = unskilled labor; 4 = own business	
Education		0 = no formal education
1 = primary; 2 = secondary; 3 = tertiary	
Electricity (=1)	0 or 1	Whether or not the individual has electricity at home	
Fishing skills	1-5	Self-declared fishing skills (Extremely good-extremely poor)	
MR depreciation		Whether or not marine resources in your local area are degrading (-1 = disagree; 0 = not sure ; 3 = Agree)	
MR sustainability		Whether or not the sustainability of marine resources is impacted by human actions (-1 = disagree; 0 = not sure ; 3 = Agree)	
Vessel ownership		Whether or not the individual owns the vessel:
1 = owned by the individual herself; 2 = partly owned
3 = crew member; 	
Crew size		Number of people in the boat crew	
IC performance		Individuals Average Performance in 4 rounds of memory task	
Vil. Pop.		The total number of people living in the village	
Tourist village (=1)	0 or 1	Whether or not the village is a major tourism hub	
Camp. village (=1)	0 or 1	Whether or not the village is a seasonal settlement	
sd_group_age		Standard deviation of age within the group	
Group trust		Level of trust within the group	
district_fish_income		The total earnings from fishing activities in the village	


Table B: Summary statistics
Variable	Mean	Std. Dev.	Min	Max	
Age	40.90	14.74	17	80	
fishing experience	21.23	13.88	1	70	
HH members	7.41	4.41	0	40	
education level	1.50	1.03	0	4	
Crew size	5.75	 7.78	0	70	
					
		Percentage			
Cell phone		87.30			
Electricity		35.71			
Transport		56.75			
Gear ownership		76.19			
Boat ownership		63.89			
Dago/Migratory fisher		27.78			
Alternative livelihoods:	None (32.94)	Farming (44.44)	Skilled worker (9.52)	Unskilled worker (11.90)	


Table C: Time pref. and Income per unit of effort from fishing 
	Ln (income per unit of effort)	
	(1)	(2)	(3)	(4)	(5)	(6)	
IDF	0.103
(0.190)	
	0.118
(0.190)	0.0790
(0.189)	-0.0287
(0.186)	0.0220
(0.203)	
Present biased (=1)	-
	-0.287*
(0.146)	-0.292**
(0.147)	-0.273*
(0.151)	-0.295*
(0.154)	-0.449***
(0.157)	
Risk averse (=1)	-
	-
	-
	-0.286*
(0.156)	-0.320**
(0.154)	-0.172
(0.151)	
Fishing skills	-
	-
	-
	-
	0.0650
(0.0781)	0.0241
(0.0791)	
age	-
	-
	-
	-
	-0.00723
(0.00487)	-0.0122**
(0.00537)	
HH members	-
	-
	-
	-
	0.00302
(0.0163)	-0.00128
(0.0191)	
Edu	-
	-
	-
	-
	0.0528
(0.0862)	0.0161
(0.0905)	
cellphone (=1)	-
	-
	-
	-
	0.430**
(0.173)	0.501***
(0.190)	
Vessel ownership (sole owner)	
Partly owned with others	-
	-
	-
	-
	-
	0.138
(0.170)	
No ownership	-
	-
	-
	-
	-
	0.185
(0.179)	
Crew size	-
	-
	-
	-
	-
	0.00441
(0.00753)	
MR depreciation	-
	-
	-
	-
	-
	-0.174*
(0.0908)	
MR sustainability	-
	-
	-
	-
	-
	0.108
(0.0860)	
Gear_Line	-	-	-	-	-	-	
Gear_Dema	-
	-
	-
	-
	-
	0.168
(0.230)	
Gear_Nets	-
	-
	-
	-
	-
	-0.0970
(0.207)	
Gear_Spear	-
	-
	-
	-
	-
	1.542**
(0.765)	
Cons.	7.905***
(0.125)	8.030***
(0.0817)	7.957***
(0.128)	8.173***
(0.174)	8.019***
(0.395)	8.204***
(0.465)	
R2	0.001	0.014	0.016	0.034	0.094	0.183	
N	188	188	188	188	188	185	
Notes: (1) OLS regression model where dependent variable is income per unit of effort from fishing activities. (2) Robust standard errors in parentheses. (3) * p < 0.1, ** p < 0.05, *** p < 0.01
Table D: time preferences and extraction in CPR experiments (random effects regression model)
	Extraction rate	
	Control groups	Time Treatment groups	
	(1)	(2)	(3)	(4)	(5)	(6)	(7)	(8)	(9)	(10)	
IDF	0.138	-	0.777	0.412	0.696	23.78**	-	29.81***	28.03***	28.79***	
	(10.81)		(10.51)	(8.906)	(9.484)	(11.07)		(9.005)	(8.353)	(8.234)	
Round number	2.468**	2.468**	2.468*	2.468*	2.468*	0.957	0.957	0.957	0.957	0.957	
	(1.258)	(1.258)	(1.262)	(1.266)	(1.267)	(1.439)	(1.439)	(1.443)	(1.448)	(1.450)	
Present biased (=1)	-	-7.684	-5.118	-5.986	-2.964	-	3.652	1.800	5.010	6.095	
		(13.12)	(13.12)	(13.37)	(14.95)		(9.907)	(10.31)	(9.419)	(9.728)	
cellphone (=1)	-	-	-20.62***	-22.25***	-19.61***	-	-	-19.98*	-29.21***	-29.43***	
			(5.107)	(4.520)	(4.373)			(10.72)	(9.259)	(9.261)	
Risk averse (=1)	-	-	-2.795	-1.540	-3.060	-	-	-12.82*	-17.62***	-18.06***	
			(7.959)	(7.098)	(7.779)			(6.967)	(5.863)	(5.880)	
Age	-	-	-	0.0160	0.114	-	-	-	-0.778***	-0.728**	
				(0.291)	(0.324)				(0.292)	(0.301)	
HH members	-	-	-	-1.832	-1.643	-	-	-	1.006	1.025	
				(1.150)	(1.168)				(0.746)	(0.715)	
Edu	-	-	-	0.142	1.945	-	-	-	-2.603	-2.087	
				(6.308)	(6.591)				(4.782)	(4.487)	
MR sustainability	-	-	-	-	-9.589	-	-	-	-	-3.690	
					(6.645)					(3.731)	
Cons.	67.51***	68.99***	86.07***	100.1***	85.44***	67.21***	81.29***	87.87***	125.2***	120.1***	
	(7.664)	(5.900)	(9.114)	(18.59)	(22.23)	(11.79)	(10.12)	(13.41)	(17.74)	(18.84)	
R2 	0.04	0.06	0.04	0.06	0.09	0.03	0.01	0.08	0.12	0.13	
No. of players	94	94	94	94	94	94	94	94	94	94	
No. of obs.	470	470	470	470	470	470	470	470	470	470	
Notes: (1) Random effects panel regression model where dependent variable is extraction rate per round. (2) Cluster robust standard errors in parentheses (3) * p < 0.1, ** p < 0.05, *** p < 0.01. (4) Column 1-5 looks at the control groups only, whereas column 6-10 look at the Time Treatment groups.


Table E: time preferences and extraction in CPR experiments (pooled OLS models)
	Extraction rate	
	Control group	Time Treatment group	
	(1)	(2)	(3)	(4)	(5)	(6)	(7)	(8)	(9)	(10)	
IDF	0.138
(5.902)	-	0.404
(5.886)	0.453
(6.044)	0.700
(6.049)	23.78***
(6.192)	-	23.61***
(6.219)	24.03***
(6.201)	22.70***
(6.043)	
Present biased (=1)	-	-7.684
(5.879)	-7.696
(5.880)	-7.710
(5.902)	-4.597
(6.401)	-	3.652
(5.355)	2.292
(5.309)	2.573
(5.182)	6.157
(5.307)	
1.Round number											
2.Round number	-0.213
(6.483)	-0.213
(6.460)	-0.213
(6.466)	-0.213
(6.474)	-0.213
(6.321)	5.745
(7.523)	5.745
(7.668)	5.745
(7.529)	5.745
(7.456)	5.745
(7.390)	
3.Round number	1.596
(7.005)	1.596
(6.964)	1.596
(6.970)	1.596
(6.976)	1.596
(6.802)	9.787
(7.530)	9.787
(7.569)	9.787
(7.524)	9.787
(7.459)	9.787
(7.334)	
4.Round number	8.936
(6.916)	8.936
(6.894)	8.936
(6.901)	8.936
(6.909)	8.936
(6.642)	5.957
(7.468)	5.957
(7.538)	5.957
(7.478)	5.957
(7.403)	5.957
(7.264)	
5.Round number	7.766
(6.901)	7.766
(6.892)	7.766
(6.900)	7.766
(6.907)	7.766
(6.698)	4.681
(7.515)	4.681
(7.604)	4.681
(7.519)	4.681
(7.499)	4.681
(7.419)	
Risk averse (=1)	-	-	-	0.183
(4.777)	-0.919
(4.792)	-	-	-	-16.10***
(5.109)	-19.40***
(5.254)	
age	-	-	-	-	0.201
(0.154)	-	-	-	-	-0.447***
(0.167)	
HH members	-	-	-	-	-1.519***
(0.493)	-	-	-	-	0.889*
(0.528)	
Edu	-	-	-	-	2.094
(2.895)	-	-	-	-	-4.302*
(2.501)	
MR sustainability	-	-	-	-	-10.97***
(2.769)	-	-	-	-	-3.264
(3.177)	
Cons.	71.29***
(5.840)	72.77***
(4.767)	72.52***
(5.920)	72.37***
(7.224)	67.18***
(11.20)	64.85***
(6.693)	78.93***
(5.782)	64.42***
(6.805)	75.57***
(7.653)	92.61***
(12.54)	
R2	0.007	0.011	0.011	0.011	0.062	0.035	0.005	0.035	0.056	0.086	
No. of players	94	94	94	94	94	94	94	94	94	94	
No. of obs.	470	470	470	470	470	470	470	470	470	470	
Notes: (1) Pooled OLS regression model where dependent variable is extraction rate per round. (2) Cluster robust standard errors in parentheses (3) * p < 0.1, ** p < 0.05, *** p < 0.01. (4) Column 1-5 looks at the control groups only, whereas column 6-10 look at the Time Treatment groups.

Table F: regression models for performance in memory task explaining extraction rates and time preferences 
	Table 5a	Table 5b	
	IDF	Extraction rate	
IC performance	0.0715***	1.883*	
	(0.0228)	(1.073)	
cellphone (=1)	0.191	-20.70***	
	(0.166)	(5.883)	
Round number		1.713*	
		(0.886)	
Time Treatment (=1)		9.140	
		(5.606)	
Cons.	-0.0877	69.57***	
	(0.240)	(10.12)	
sigma			
Cons.	0.838***		
	(0.0868)		
R2	0.03	0.05	
No. of players	188	198	
No. of obs.	188	940	
Notes: 
˗	Table 5(a) tobit regression model with IDF as the main dependent variable, Table 5(b) random effects panel regression model where dependent variable is extraction rate per round. 
˗	R2 in Column 1 is pseudo R2 , whereas R2 for the panel model in Column 2 is the R2 for overall variation (both within and between)
˗	Robust standard errors in parentheses.  
˗	 * p < 0.1, ** p < 0.05, *** p < 0.01. 


Table G: Investments and time preferences
	Ln (investment)	
	(1)	(2)	
IDF	1.074**
(0.540)	1.067**
(0.502)	
Present biased (=1)	0.723
(0.515)	0.216
(0.409)	
Risk averse (=1)	0.0573
(0.392)	-0.0543
(0.347)	
Age	0.00218
(0.0152)	-0.0124
(0.0124)	
HH members	0.0284
(0.0480)	0.0380
(0.0417)	
Edu.	0.0955
(0.263)	0.0675
(0.217)	
Fishing skills	0.136
(0.222)	0.126
(0.180)	
Vessel ownership (sole owner)	
Partly owned with others	-0.510
(0.496)	-0.299
(0.440)	
No ownership	-0.520
(0.670)	-0.476
(0.570)	
Crew size	0.148***
(0.0445)	0.162***
(0.0451)	
cellphone (=1)	-0.440
(0.535)	-0.747*
(0.451)	
MR sustainability	-0.103
(0.276)	-0.124
(0.226)	
MR depreciation	0.226
(0.271)	0.276
(0.232)	
Cons.	10.23***
(1.283)	11.03***
(1.091)	
R2	0.179	0.178	
N	122	160	
Notes: 
˗	OLS regression model where dependent variable is investment on fishing equipment 
˗	Column 1 IDF is based on average of two lists for only consistent players; For Column 2 IDF is the average of first switching point for both lists, and therefore includes inconsistent players. 
˗	Robust standard errors in parentheses. 
˗	 * p < 0.1, ** p < 0.05, *** p < 0.01.


Robustness checks 

Table H: Robustness check 1-Income & time preferences
	Ln (income per unit of effort)	
	(1)	(2)	(3)	(4)	(5)	
IDF	0.0301
(0.202)	0.0331
(0.170)	0.0465
(0.178)	0.0583
(0.177)	0.0583
(0.147)	
Present biased (=1)	-0.423**
(0.163)	-0.441**
(0.166)	-0.283*
(0.144)	-0.265*
(0.143)	-0.265*
(0.138)	
Risk averse (=1)	-0.194
(0.159)	-0.189
(0.122)	-0.194
(0.137)	-0.189
(0.134)	-0.189*
(0.0957)	
Fishing skills	-0.0191
(0.0829)	-0.0298
(0.106)	-0.0486
(0.0638)	-0.0601
(0.0636)	-0.0601
(0.0787)	
Age	-0.0167***
(0.00587)	-0.0184***
(0.00508)	-0.00985**
(0.00462)	-0.0112**
(0.00494)	-0.0112**
(0.00390)	
Alternate livelihood						
Farming	0.128
(0.168)	0.0890
(0.224)	0.0149
(0.142)	-0.00988
(0.158)	-0.00988
(0.141)	
Unskilled labor	-0.360
(0.245)	-0.394*
(0.194)	-0.218
(0.219)	-0.257
(0.224)	-0.257
(0.177)	
Skilled labor	-0.201
(0.270)	-0.251
(0.277)	-0.242
(0.220)	-0.255
(0.218)	-0.255
(0.235)	
Others	-0.0316
(0.343)	-0.0941
(0.504)	0.277
(0.330)	0.184
(0.363)	0.184
(0.334)	
Edu	-0.0502
(0.0990)	-0.0362
(0.122)	-0.0201
(0.0794)	0.0000819
(0.0760)	0.0000819
(0.0956)	
Electricity (=1)	0.230
(0.173)	0.239
(0.189)	0.157
(0.137)	0.156
(0.138)	0.156
(0.134)	
Dago fisher (=1)	-0.237
(0.195)	-0.208
(0.232)	-0.164
(0.153)	-0.152
(0.156)	-0.152
(0.153)	
cellphone (=1)	0.462**
(0.184)	0.421**
(0.176)	0.408**
(0.165)	0.382**
(0.174)	0.382**
(0.139)	
Vessel ownership (sole owner)	
Partly owned with others 	0.0835
(0.169)	0.104
(0.162)	0.0735
(0.152)	0.0902
(0.149)	0.0902
(0.146)	
No ownership	0.146
(0.181)	0.159
(0.163)	0.0967
(0.153)	0.109
(0.147)	0.109
(0.147)	
Crew size	0.00387
(0.0117)	0.0101
(0.00953)	-0.00111
(0.0103)	0.00571
(0.00890)	0.00571
(0.00880)	
MR depreciation	-0.187**
(0.0908)	-0.189
(0.117)	-0.170**
(0.0779)	-0.167**
(0.0767)	-0.167*
(0.0876)	
MR sustainability	0.0925
(0.0856)	0.0990
(0.0787)	0.0277
(0.0741)	0.0283
(0.0730)	0.0283
(0.0772)	
Gear_Line						
Gear_Dema	0.237
(0.263)	0.261
(0.323)	0.326
(0.199)	0.361*
(0.200)	0.361
(0.205)	
Gear_Nets	-0.0274
(0.228)	-0.00943
(0.307)	-0.00344
(0.185)	0.0105
(0.180)	0.0105
(0.248)	
Gear_Spear	1.546*
(0.788)	1.511
(0.865)	0.785
(0.520)	0.781
(0.508)	0.781
(0.565)	
Vil. pop.	-
	-0.0000231
(0.0000193)	-
	-0.0000245
(0.0000271)	-0.0000245
(0.0000189)	
Camp. village (=1)	-
	0.00907
(0.147)	-
	0.0359
(0.244)	0.0359
(0.105)	
Tourist village (=1)	-
	0.231
(0.135)	-
	0.266*
(0.151)	0.266
(0.157)	
Cons.	8.561***
(0.503)	8.622***
(0.502)	8.319***
(0.437)	8.310***
(0.442)	8.310***
(0.456)	
District dummies	Yes	No	Yes	No	No	
R2	0.235	0.235	0.164	0.159	0.159	
N	185	185	238	238	238	
Notes: 
˗	OLS regression models where dependent variable is income per unit of effort from fishing activities 
˗	Column 1 & 2 IDF is based on average of two lists for only consistent players; For Column 3,4 & 5 IDF is the average of first switching point for both lists, and therefore includes inconsistent players. 
˗	Column 1, 3 and 4 robust standard errors, Column 2 & 5 standard errors clustered at the village level. 
˗	 * p < 0.1, ** p < 0.05, *** p < 0.01.


Table I: Robustness check 2-CPR & time preferences
	Extraction in 1st round	Extraction rate (Round 2-5)	
	(1)	(2)	(3)	(4)	
	Control Group	Time Treatment Group	Control Group		Time Treatment Group	
IDF	14.84
(10.90)	25.60**
(10.18)	2.775
(7.943)	24.83***
(7.544)	
Present biased (=1)	-12.93
(17.23)	12.82
(11.35)	-6.501
(15.98)	3.096
(10.42)	
Risk averse (=1)	-6.942
(8.164)	-12.23
(10.64)	-3.555
(8.153)	-15.97**
(6.844)	
Age	0.713*
(0.367)	-1.008*
(0.533)	0.233
(0.362)	-0.616**
(0.299)	
HH members	-2.410*
(1.236)	0.753
(0.967)	-1.428
(1.162)	1.147*
(0.697)	
Edu	5.808
(8.640)	-3.133
(4.619)	3.123
(6.732)	3.296
(3.304)	
cellphone (=1)	-25.96***
(7.891)	-39.66**
(14.97)	-11.57**
(4.866)	-22.98**
(10.49)	
MR sustainability	-13.19*
(6.677)	-9.415**
(4.401)	-6.887
(6.605)	-4.314
(2.853)	
Round number	-
	-
	2.075
(1.540)	-1.129
(1.393)	
Group extr. (t-1)	-
	-
	0.0619***
(0.0199)	0.0339***
(0.0112)	
Cons.	57.36**
(27.08)	165.9***
(29.53)	47.10*
(27.78)	119.0***
(27.04)	
District dummies	Yes	Yes	Yes	Yes	
R2	0.225	0.262	0.19	0.24	
No. of players	94	94	94	94	
No. of obs.	94	94	376	376	
Notes: 
˗	Column 1 & 2 OLS regression model where dependent variable extraction rate in the first round, Column 3&4 Random effects panel regression model where dependent variable is extraction rate per round 
˗	Cluster robust standard errors in parenthesis. 
˗	 * p < 0.1, ** p < 0.05, *** p < 0.01.


Table J: Robustness check 3- CPR & time preferences
	Extraction rate	
	Control groups	Time Treatment groups	
	(1)	(2)	(3)	(4)	(5)	(6)	(7)	(8)	(9)	(10)	
											
IDF	3.877
(5.607)	5.247
(5.652)	1.190
(3.525)	2.897
(10.81)	2.313
(10.04)	25.60***
(5.780)	30.74***
(5.217)	16.86**
(7.361)	28.19***
(9.454)	21.73**
(9.480)	
Present biased (=1)	-6.321
(20.38)	-5.829
(21.21)	-0.234
(17.12)	-6.075
(9.870)	-1.022
(8.710)	5.648
(6.775)	7.435
(7.723)	1.628
(8.699)	1.203
(8.198)	4.646
(7.282)	
Round number	2.468*
(1.330)	2.468*
(1.331)	2.175*
(1.239)	2.468**
(0.981)	2.175**
(0.881)	0.957
(1.062)	0.957
(1.063)	2.025
(1.307)	0.957
(1.146)	2.025*
(1.045)	
Risk averse (=1)	-4.963
(8.383)	-1.467
(8.198)	-6.870
(8.135)	-4.902
(8.549)	-6.358
(7.286)	-16.18**
(6.960)	-6.296
(6.725)	-7.723*
(4.412)	-14.58*
(7.485)	-5.530
(6.692)	
age	0.269
(0.441)	0.282
(0.430)	0.0722
(0.344)	0.370
(0.286)	0.116
(0.238)	-0.778**
(0.303)	-0.408
(0.332)	-0.317
(0.238)	-0.707**
(0.280)	-0.136
(0.265)	
HH members	-1.695
(1.421)	-1.921
(1.522)	-2.018**
(1.001)	-1.912*
(1.049)	-1.906**
(0.894)	1.126
(0.927)	1.263*
(0.709)	0.965
(0.983)	1.116*
(0.655)	1.259*
(0.648)	
Edu.	3.069
(6.736)	4.051
(6.508)	0.768
(4.341)	6.098
(4.834)	2.421
(3.998)	1.699
(3.112)	2.830
(2.586)	-0.284
(3.714)	5.479
(4.266)	2.018
(4.104)	
MR sustainability	-9.650
(7.568)	-9.774
(7.546)	-8.662
(5.742)	-9.893**
(4.747)	-8.238**
(4.171)	-5.677*
(3.007)	-3.552
(3.276)	-2.389
(2.729)	-5.118
(4.408)	-2.932
(3.948)	
cellphone (=1)	-17.78***
(4.362)	-16.21***
(3.471)	-24.04***
(4.128)	-14.03
(9.425)	-22.22***
(8.287)	-27.27***
(8.962)	-33.72***
(7.608)	-13.22
(10.55)	-29.13***
(9.077)	-15.32*
(8.223)	
Vil. Pop.	-
	-0.0000862
(0.00177)	-
	0.000932
(0.00187)	0.000489
(0.00177)	-
	0.000292
(0.00144)	-
	0.00455***
(0.00151)	0.00269*
(0.00163)	
Tourist village (=1)	-
	-5.471
(11.53)	-
	-16.57
(12.44)	-5.856
(10.68)	-
	14.44
(11.65)	-
	-8.527
(10.95)	0.388
(10.93)	
Camp village (=1)	-
	18.03
(11.91)	-
	26.35**
(10.94)	16.27*
(9.855)	-
	1.047
(5.832)	-
	13.73
(9.008)	6.065
(8.913)	
Group trust	-
	0.630
(3.771)	-
	-
	-1.155
(2.758)	-
	1.855*
(1.094)	-
	-
	2.615
(2.261)	
sd_group_age	-
	0.761
(1.346)	-
	-
	0.984
(0.955)	-
	-4.747***
(0.568)	-
	-
	-3.172***
(0.946)	
district_fish_income	-
	
	-
	2.735
(1.730)	1.351
(1.554)	-
	
	-
	5.677***
(1.468)	1.735
(1.526)	
cons	78.41***
(25.49)	56.61
(42.49)	99.18***
(20.00)	39.30
(30.17)	67.01**
(29.24)	142.5***
(21.55)	139.4***
(25.46)	93.06***
(13.97)	60.96**
(24.18)	72.92***
(27.51)	
Village cons.	-
	-
	-
	-10.59
(.)	-20.92
(821.1)	-
	-
	-
	-15.08
(13.99)	-11.74
(592.7)	
District cons.	-
	-
	-
	3.471***
(0.0859)	3.463***
(0.0766)	-
	-
	-
	3.267***
(0.119)	3.316***
(0.0876)	
lnsig_e cons.	-
	-
	-
	3.404***
(0.0365)	3.418***
(0.0323)	-
	-
	-
	3.560***
(0.0365)	3.589***
(0.0323)	
District dummies	Yes	No	Yes	No	No	Yes	No	Yes	No	No	
R2											
No. of players	94	94	120	94	120	94	94	120	94	120	
No. of obs.	470	470	600	470	600	470	470	600	470	600	
Notes: 
˗	Column 1-3 & Column 6-8 Random effects panel regression model where dependent variable is extraction rate per round, Column 4-5 & Coloumn 9-10 Mixed effects regression model where dependent variable is extraction rate per round 
˗	Column 1,2,4 & Column 5,6,9 IDF is based on average of two lists for only consistent players; For Column 5 & Column 10, IDF is the first switching point for both lists, and therefore includes inconsistent players. 
˗	Column 1-3 & Column 6-8 standard errors clustered at the village level, others clustered robust standard errors at the group level. 
˗	 * p < 0.1, ** p < 0.05, *** p < 0.01.


Table K: Control vs Treatment groups

	Control groups	Time Treatment groups	p-value (two sided t-test)	
IDF	0.64	0.63	0.83	
Present biased (=1)	0.18	0.23	0.37	
IC_performance	8.8	9.2	0.288	
Cellphone (=1)	0.88	0.88	1	
electricity (=1)	0.31	0.39	0.22	
MR sustainability	-0.48	-0.58	0.41	
MR depreciation	0.61	0.59	0.855	
Crew size	5.66	6.9	0.33	
risk	0.65	.71	0.35	
trust	0.55	0.57	0.77	
Dago (migratory) fishers (=1)	0.27	0.32	0.42	
