# Supplementary material for: Time Preferences and Natural Resource Extraction Behavior: An Experimental Study from Artisanal Fisheries in Zanzibar
Source: PLoS One. 2016 Dec 29;11(12):e0168898. doi: 10.1371/journal.pone.0168898 (PMC5199085; doi:10.1371/journal.pone.0168898)
Supplement: S2 File — (DOCX) [file pone.0168898.s002.docx]

# CPR experiment

This is a research exercise to understand how fishers manage fisheries/marine resources in this area. In this activity we try to recreate a situation where different individuals take decisions about how much to extract from local fisheries resource.

You share a small fishing site with 6 other people. This fishing site has fish stock worth about 2000 Tokens. You have to decide the number of hours to spend fishing in this area. This could be thought as the number of hours spent working on fisheries extraction related activities per day. Remember the decision you take will determine how much you earn during the course of experiments, so take your time and make your decision after thinking carefully. The money you earn during the experiment comes from university. These earnings will be delivered to you in terms of cellphone credit. The assistants will give you a phone number which you can contact if you don´t receive the money on the given day. At the end of the session you will be given a receipt of your earnings (which carries the amount and date you receive your earnings). Please do not forget to sign this receipt and give it back to the assistants (you can keep your copy of the receipt). You are free to leave at any point during the experiment, however if you decide to leave before the end of the experiment you will not be paid.

You should listen to the instructions very carefully and ask questions at any point. We will conduct short quizzes to see if you understand the experiment. You can only participate in the experiment when you fully understand it.

It is important to remember that decisions taken in the experiment are absolutely individual, you don´t have to share them with any one. To ensure that you can make decisions independently, players in the same group are to be seated in such a way that no one is able to see other player´s decision.

## For Assistants only

You will be divided into teams of two. First team will carry out treatment T_0_. Second team will carry out treatments T_1_. I will tell you at the start of each day which treatment you have to carry out.

## Tables

| G_1_ (e, z) | | |
| --- | --- | --- |
| effort | reward | destruction |
| 0 | 0 | 0 |
| 2 | 20 | 30 |
| 4 | 40 | 60 |
| 6 | 60 | 90 |
| 8 | 80 | 120 |
| 10 | 100 | 150 |
| 12 | 120 | 180 |
| 14 | 140 | 210 |
| 16 | 160 | 240 |

## The decisions form

Each participant will receive the decision form before the start of the experiment. On top right corner of this form you will see a blank space. You should write the player ID number you were given at the start of experiment in this blank space. One of the assistants will come to you to see if you have written your ID number.

You should write the number of hours you want to devote to fishing activities in the given blank space (assistants demonstrate where to write the number of hours). Once you are done, an assistant will come to you to collect your decision form.

## For team carrying out T_0_

### Decision scenario A (Activity A)

Table 1 show you the net worth of fish you can take home for the amount of hours worked. So for example if you work for 2 hours you get 20 tokens worth of fish plus the amount that is left in the shared pool. However you should also note that working for 2 hours means that you destroy 30 tokens work of fish stock.

As you can see the more you work the more you can earn, however also the more you work the more you destroy the fishing site and so the share of income from this fishing site decreases.

Your total earnings in one round depend upon:

1. The number of hours you worked (extraction earnings)
2. The worth of fish stock which is not extracted and is divided equally among all group members (conservation earnings) (for time treatment group mention that this will be delivered 14days after the experiment)

Remember we calculate the total destruction caused by the whole group (i.e. adding each member’s destruction), which is then subtracted from 2000 tokens. This amount is to be divided equally between the group members (for time treatment group mention that this will be delivered 14days after the experiment).. You will be made aware of the total destruction caused by the group at the end of the round.

[Example 1: extreme case no extraction]

Consider for example if none of you do any fishing, then at the end of the round this 2000 TOKENS is divided equally between all of you (for time treatment group mention that this will be delivered 14days after the experiment).

[Example 2: extreme case maximum extraction by all]

Consider for example the case where each player works for the maximum amount. This means (depending on the gear) that the total destruction caused by the group is 240 * 6 = 1440. We subtract this amount from the original endowment (2000-1440 = 560). This 560 is then divided equally between all group members (560/6 = 93 per player) (for time treatment group mention that this will be delivered 14days after the experiment). So each player gets 92 + 160 = 253.

[Example 3: mixed case maximum extraction only by you]

Consider for example the case where only one player works for the maximum amount and the rest do not work. This means (depending on the gear) that the total destruction caused by the group is 240 * 1 = 240. We subtract this amount from the original endowment (2000-240 = 1760). This 1760 is then divided equally between all group members (1760/6 =2 93 per player) (for time treatment group mention that this will be delivered 14days after the experiment). The person who worked gets 160 + 293 = 453 while all others get 293.

[Example 4: mixed case different extraction levels]

Consider for example the case where 2 players work for 2 hours and the rest (4 players) work 4 hours. This means that the total destruction caused by the whole group is 30+30+60+60+60+60 = 300. We subtract this amount from the original endowment (2000-300 = 1700). This 1700 is then divided equally between all group members (1700/6 = 283) (for time treatment group mention that this will be delivered 14days after the experiment).

In this case the players who worked for 2 hours earned:

20 + 283 = 302 TOKENS

Whereas those worked for 4 hours earned:

40 + 283 = 322 TOKENS

## For team carrying out T1

### Decision scenario A (Activity A)

Table 1 show you the net worth of fish you can take home for the amount of hours worked. So for example if you work for 2 hours you get 20 tokens worth of fish plus the amount that is left in the shared pool. However you should also note that working for 2 hours means that you destroy 30 tokens work of fish stock.

As you can see the more you work the more you can earn, however also the more you work the more you destroy the fishing site and so the share of income from this fishing site decreases.

Your total earnings in one round depend upon:

1. The number of hours you worked (extraction earnings)
2. The worth of fish stock which is not extracted and is divided equally among all group members (conservation earnings) (for time treatment group mention that this will be delivered 14days after the experiment).

Remember we calculate the total destruction caused by the whole group (i.e. adding each member’s destruction), which is then subtracted from 2000 tokens. This amount is to be divided equally between the group members. You will be made aware of the total destruction caused by the group at the end of the round.

[Example 1: extreme case no extraction]

Consider for example if none of you do any fishing, then at the end of the round these 2000 tokens are divided equally between all of you (for time treatment group mention that this will be delivered 14days after the experiment).

[Example 2: extreme case maximum extraction by all]

Consider for example the case where each player works for the maximum amount. This means (depending on the gear) that the total destruction caused by the group is 240 * 6 = 1440. We subtract this amount from the original endowment (2000-1440 = 560). This 560 is then divided equally between all group members (560/6 = 93 per player) (for time treatment group mention that this will be delivered 14days after the experiment). So each player gets 92 + 160 = 253.

[Example 3: mixed case maximum extraction only by you]

Consider for example the case where only one player works for the maximum amount and the rest do not work. This means (depending on the gear) that the total destruction caused by the group is 240 * 1 = 240. We subtract this amount from the original endowment (2000-240 = 1760). This 1760 is then divided equally between all group members (1760/6 =2 93 per player) (for time treatment group mention that this will be delivered 14days after the experiment). The person who worked gets 160 + 293 = 453 while all others get 293.

[Example 4: mixed case different extraction levels]

Consider for example the case where 2 players work for 2 hours and the rest (4 players) work 4 hours. This means that the total destruction caused by the whole group is 30+30+60+60+60+60 = 300. We subtract this amount from the original endowment (2000-300 = 1700). This 1700 is then divided equally between all group members (1700/6 = 283) (for time treatment group mention that this will be delivered 14days after the experiment).

In this case the players who worked for 2 hours earned:

20 + 283 = 302 tokens

Whereas those worked for 4 hours earned:

40 + 283 = 322 tokens

## Summary of instructions for CPR experiment

1. Using the payoff table decide how many hours do you want to devote to fishing activities
2. In the decision form write your decision;
3. Hand the decision form to one of the assistants
4. Wait for the assistant to calculate the total destruction caused by all the extraction in the group. The assistant will announce the level of resource destroyed publicly
5. One of the assistant will come to you and explain to you how much money you earned by extraction and how much money you earned from conservation

# Time preference task

## Experiment Design

In this study you will make decisions between two options. The ﬁrst option will always be called OPTION A. The second option will always be called OPTION B. Each decision you make is a choice. For each decision, all you have to do is decide whether you prefer OPTION A or OPTION B.

These decisions will be made in 2 separate blocks of tasks. Each block of tasks is slightly different, and so new instructions will be read at the beginning of each task block. Once all of the decision tasks have been completed, we will randomly select one decision as the decision-that-counts. Each decision has an equal chance of being the decision-that-counts. If you preferred OPTION A in the decision-that-counts, then OPTION A would be implemented. If you preferred OPTION B, then OPTION B would be implemented decision-that-counts.

|  | T1 (start date) | Option A | T2 (later date) | Option B |
| --- | --- | --- | --- | --- |
| 1 | 0 | 3500 | 14 | 4000 |
| 2 | 0 | 2700 | 14 | 4000 |
| 3 | 0 | 2000 | 14 | 4000 |
| 4 | 0 | 1200 | 14 | 4000 |
| 5 | 0 | 500 | 14 | 4000 |

| 6 | 1 | 3500 | 15 | 4000 |
| --- | --- | --- | --- | --- |
| 7 | 1 | 2700 | 15 | 4000 |
| 8 | 1 | 2000 | 15 | 4000 |
| 9 | 1 | 1200 | 15 | 4000 |
| 10 | 1 | 500 | 15 | 4000 |

**Risk preference experiments**

In this task you will make a number of choices between two fishing alternatives named A and B.

There is no correct answer. We are only interested in your choices. We acknowledge that these choices are not perfectly equal to real-life fishery choices, but we are very interested in your judgment.

You will be presented with two different options. You have to choose between one of them.

These two options could be thought as two different fishing trips. The return of these fishing trips depends on the weather conditions (good or bad). If the weather is good you get more money and if the weather is bad you get less money. The probability of having good weather is 0.50 (same as bad weather).

Tell participants that they would be presented with 10 different cases.

- In each case they have to decide whether to choose Option A or Option B
- Option A and option B differ in terms of the amount of money and the risk associated with the amounts (make reference to weather conditions)
- So for example in 1^st^ case,
  - If they chose option A this means there is 50% probability that they get 50 TSH and 50% probability that they get 4950 TSH. Whether they are paid 50 TSH or 4950 TSH depends on coin toss
  - On the other hand if they chose option B this means there is 50% probability that they get 2650 TSH and 50% probability that they get 2750 TSH. Whether they are paid 2650 TSH or 2750 TSH depends on coin toss
- The values of Option A and Option B changes for one case to another
- Remind them that they would be paid on one randomly selected case. Tell them that it is important they think about each case very carefully as their payment could be any one of the 10 cases

**Remember**

Participants should know that the coin toss would only take place after they had made their choice in all the 10 cases and that this coin toss would only be for the chosen decision

**Table**

|  | Option A (TSH) | |  | Option B (TSH) | |
| --- | --- | --- | --- | --- | --- |
|  | p = 0.5 | 1- P = 0.5 |  | p = 0.5 | 1 - P = 0.5 |
| 1 | 50 | 4950 |  | 2650 | 2750 |
| 2 | 1100 | 3900 |  | 2650 | 2750 |
| 3 | 2400 | 2600 |  | 2650 | 2750 |
| 4 | 2400 | 2600 |  | 2000 | 3400 |
| 5 | 2400 | 2600 |  | 1900 | 3500 |
| 6 | 2400 | 2600 |  | 1750 | 3650 |
| 7 | 2400 | 2600 |  | 1600 | 3800 |
| 8 | 2400 | 2600 |  | 1450 | 3950 |
| 9 | 2400 | 2600 |  | 1050 | 4350 |
| 10 | 2400 | 2600 |  | 200 | 5200 |

**Memory task**

It should be noted that memory task was done as a part of experiment on individuality and competitiveness, whereby participants had to declare whether they want to receive their earnings based on competitive basis or not. However in order to establish baseline behavior we played one round (always the first round) where participants did not have to make any choice. We use performance in this first round as our measure of cognitive ability.

For the purpose of this paper we only focus on the memory related part of the task. Detailed experimental protocols are available upon request.

## Step 1: Explain the game

- First explain that they would be shown a page with pictures of 25 different things. And that they would be asked to recall these images afterwards. They would get 2-3 minute to see the pictures and 2-3 minutes to remember
- Show the participants Sample sheet
- Tell the participants that there would be multiple rounds of this task, and they would be paid based on their performance and that choices made in1 randomly selected round will determine their earnings. As this chosen round could be any one so they should think about their choices in each round very carefully

## Step 2: Predictions about Relative and absolute performance

- Ask the participants to predict “How well you can perform the task i.e. how many pictures they can remember (from 0 to 25)”
  - Note this “Average performance” on the Individuality Experiment-Form
  - Remind them that if they get this correct they would get extra 200 TZS, so they should answer carefully
- Also ask the participants to rank their skill in performing the task.
  - *Scale:* The Best, Better than most, Average, worse than most, very poor (the worst) or their position (1 to 6) whichever they prefer.
  - Note this “Rank” on the Experiment-Form

## Step 3: Start with the first round of the activity
