# Supplementary material for: Time Preferences and Natural Resource Extraction Behavior: An Experimental Study from Artisanal Fisheries in Zanzibar
Source: PLoS One. 2016 Dec 29;11(12):e0168898. doi: 10.1371/journal.pone.0168898 (PMC5199085; doi:10.1371/journal.pone.0168898)
Supplement: S3 File — (DOCX) [file pone.0168898.s003.docx]

**Jina la Anayehoji:** ___________________ **Jina la Anayehojiwa (ID):** _________________

**Kijiji:** ________________________ **Tarehe:**  _______________________

# Taarifa za kaya

| 1. Umri | 1.2 Elimu | 1.3 ajira ya kipato muhimu | 1.4 Ajira ya kipato cha pili | 1.5 Idadi ya wana kaya | 1.6 Idadi ya wanamtegemea (Umri chini ya 18) (Umri Zaidi ya 60) | |
| --- | --- | --- | --- | --- | --- | --- |
|  |  |  |  |  |  |  |
|  |  |  |  |  |  |  |

### Wavuvi wahamiaji

1. Kijiji unachotoka? __________________________
2. Kwa nini ulihamia ______________________
3. Je ni kwawaida yako kuhama hama kaita sehemu mbali? Ndio Hapana

# Taarifa za uvuvi

| 1. # ya miaka katika kazi ya uvuvi | 5.2 Unamiliki chombo miliki/ni mmoja katikakundi la wavuvi | 5.3 Aina ya chombo | 5.4 Jumla ya idadi ya wavuvi |
| --- | --- | --- | --- |
|  |  |  |  |

5.3 Key: 1.Canoe; 2.Outrigger canoe; 3.Dhow; 4.Boat (Mashua); 5.Dinghy; 6 = Ngwanda

1. Taja zana za uvuvi ulizowahi kutumia katika maika 5/10 years?

| 6.1 Zana | 6.2 Umiliki | 6.3 Samaki unaowatega | 6.4 Msimu (Normal/Off) | 6.5 Ujuzi wa kutumia zana hii (Miaka) | 6.6 Idadi ya wavuvi | 6.7 wingi wa samaki unaokamata |
| --- | --- | --- | --- | --- | --- | --- |
|  |  |  |  |  |  |  |
|  |  |  |  |  |  |  |
|  |  |  |  |  |  |  |
|  |  |  |  |  |  |  |
|  |  |  |  |  |  |  |

1. Mara ngapi unabadili zana? (kila mwezi/msimu/mwaka/sibadili)

______________________________________________________________________

1. Je zana hii ndio unazozipenda Ndio Hapana
2. Taja zana unazozipenda? (dema, mikuki, Nyavu)____________________________
3. Je umebadili zana unayoitumia katika miaka 5 iliyopita? Ndio Hapana
4. WKitu gani kilikufanya ubadili zana?________________________________
5. Nani kakuanzisha utumiaji wa zana hii? ______________________________________________

### Kama wanamiliki zana zao wenyewe (kama sivyo endelea na masuala yanayofuatia)

1. Umetumia kiasi gani (au gharama) kwa zana zu uvuvi kipindi klichoptia? ________________________
2. Gharama za ujumla kwa:

Kununua zana _____________________________________________________________

Matumizi ya zana hii kwa siku na kwa mwezi ________________ Matengenezo_____________________

1. Gharama hizi zimebadilika ukilinganisha na mwaka jana? (Imezidi/Pungua/Imebaki vile vile)

________________________________________________________________________

1. Kwa wastani siku ngapi ulikwenda kuvua katika mwezi(ukizingatia mwaka 2012-3)?

Msimu mzuri: _______________________ Msimu wa kawaida: ___________________________

Nje ya msimu: ________________________

1. Je unagawana samaki uliovua na wavuvi wenzako? Ndio Hapana

### Ametumwa na mtu mwengine kuifanya hiyo kazi

1. Ulikuwa unatumia zana gani kabla ya kuungana na hawa wavuvi wenzako? _______________________________
2. Mara ngapi unabadili boti au kuvua na wavuvi wengine? ________________________________________
3. Je ulikuwa unawaelewa mwenye kiti na kamati ya uvuvi/kepteni wa chombu au baadhi ya wavuvi unaovua nao kable ya kujiunga? ____________________________________________________________________
4. Kwa wastani siku ngapi ulikwenda kuvua katika mwezu (2012-3)?

Msimu mzari: _______________________ Msimu wa kawaida: ___________________________

Nje ya msimu: ________________________

1. Aslimia ngapi ya samaki walivuliwa hugawanywa kwa wavuvi waliomo katika chombo? ____________________________________

### Samaki waliovuliwa

1. Taja wastani wa kipato unachopata katika kila siku unayokwenda kuvua? _______________________________________

Msimu mzari: _______________________ Msimu wa kawaida: ___________________________

Nje ya msimu: ________________________

1. In your view, has your income from fishing increased, remained stable or decreased in the last year? ___________________________________________________________________
2. Wastani wa muda unaotumika kuvua katika kila siku:

Msimu mzari: _______________________ Msimu wa kawaida: ___________________________

Nje ya msimu: ________________________

1. Mwaka jana uöiwahi kuppiga dago nje ya kijiji chako? Ndio Hapana
2. Unweza kusema nini juu ya uwezo wako wa shughuli hizi za baharini?

| Uvuvi | Kuzamia(gesi,viatu,hivi hivi) | Kuogolea |
| --- | --- | --- |
|  |  |  |

1= Nzuri sana; 2= Nzuri; 3= Wastani i (hivy hivyo); 4= Mbaya; 5 = Mbaya sana

### Knowledge/opinions about gears

1. Ipi katika aina hizi ya zana za kuvulia wewe binafisi umewahi kutumia au mtu yoyote unayemfahamu ametumia katika miaka 5 iliyopita?

|  | Dema/Towe | Uzio/wando | Mkuki/Kijiti | Nvayu aina ya jarife | Cast nets | Nvayu za kukokota | Nvayu za kuzungusha | Mshipi | Bunduki | Longlines (Kaputi) |
| --- | --- | --- | --- | --- | --- | --- | --- | --- | --- | --- |
| Mwenyewe |  |  |  |  |  |  |  |  |  |  |
| Familia |  |  |  |  |  |  |  |  |  |  |
| Rafiki |  |  |  |  |  |  |  |  |  |  |

1=Ndio; 0=Hapana

#### Sababu muhimu ziliyokufanya ubadilishe aina ya zana ya kuvulia

1. Unapoamua kutumia zana ya kuvulia kitu gani kinakufanya wewe ununue ile zana. Hapo chini weka namba kwa umuhimu wake 1 mpaka 4:

Gharama

Uharibifu baharini

Kipato cha samaki

Ujuzi unaohitajika katika kutumia hiya zana

# Kipato cha kijamii

1. Idadi ya watu unao waelewa katika kikundi hiki kwa jina (katika hawa waliopo 12) __________________ Rafiki_________________ Familia _____________

*Scale:* 1 = Nakubaliana sana, 2 = Nakubali, 3 = Sina hakika, 4 = Sikubali, 5 = Sikubali kabisa

1. Nwaamini watu katika kundi hili kwa zangu __________________________
2. Kawaid naawaaamini watu wenye __________________________

## Uwezo wa kipato

1. Unao umeme nyumbani? Ndio Hapana

Unavyo vifaa hivi:

| TV | VCR/DVD | Friji/jokofu |
| --- | --- | --- |
| Feni | Radio/cassette player | Simu ya mkononi |

Njia ya kujipatia mwangaza:

| Sina | kibatari | Taa ya karabai | Umeme |
| --- | --- | --- | --- |

Usafiri

| Beskeli | Gari | Pikipiki | Nyengineyo: |
| --- | --- | --- | --- |

Uwezekaji wa paa umetumia?

| makuti | Bati | Tiles | Nyengineyo: |
| --- | --- | --- | --- |

Sakafu

| Udongo | Mbao | Saruji | Tiles |
| --- | --- | --- | --- |

1. **Cell phone Questionnaire**
2. Nani unafanya mazungumzo nae sana kwa simu yako? Kaka/Rafiki/ familia ? _________________________________
3. Kwa kiasi gani unatumia simu kwa siku?

Very frequent user frequent user Use sometimes not a lot very rarely

1. Unatumia kiasi gani kwa kupiga simk wasiku/wiki/mwezi? ________________
2. Je unahisi simu ni muhimu sana kwako?

Ndio kila siku Ndio kwa baadhi ya wakati Hapana situmii sana Situmii kabisa

1. Je unahisi simu umefanya maisha yawe rahisi kwako? Hapana Ndio
2. Je unatumia huduma ya EazyPesa/tigopesa? Hapana Ndio
