# Supplementary material for: Time Preferences and Natural Resource Extraction Behavior: An Experimental Study from Artisanal Fisheries in Zanzibar
Source: PLoS One. 2016 Dec 29;11(12):e0168898. doi: 10.1371/journal.pone.0168898 (PMC5199085; doi:10.1371/journal.pone.0168898)
Supplement: S4 File — (DOCX) [file pone.0168898.s004.docx]

**Interviewer:** ________________________ **Interviewee (ID):** _________________

**Village:** ________________________ **Date:**  _______________________

# About the household

| 1. Age | 1.2 Education | 1.3 Main source of income | 1.4 Secondary source of income | 1.5 Number of HH members | 1.6 Number of dependents  (less than 18) (greater than 60) | |
| --- | --- | --- | --- | --- | --- | --- |
|  |  |  |  |  |  |  |
|  |  |  |  |  |  |  |

### Migratory fishermen

1. What is your native village? __________________________
2. Why did you migrate ______________________
3. Do you migrate regularly to different areas? Yes No

# Fishing data

| 1. # of years as fishermen | 5.2 Do you own a vessel  Owner/part of the crew | 5.3 Type of boat | 5.4 Crew size |
| --- | --- | --- | --- |
|  |  |  |  |

5.3 Key: 1.Canoe; 2.Outrigger canoe; 3.Dhow; 4.Boat (Mashua); 5.Dinghy; 6 = Ngwanda

1. List the gears that you have used in the last 5/10 years?

| 6.1 Gears | 6.2 Ownership | 6.3 Target species catch | 6.4 Season (Normal/Off) | 6.5 Experience with this gear (years) | 6.6 Crew size | 6.7 Catch/Income |
| --- | --- | --- | --- | --- | --- | --- |
|  |  |  |  |  |  |  |
|  |  |  |  |  |  |  |
|  |  |  |  |  |  |  |
|  |  |  |  |  |  |  |
|  |  |  |  |  |  |  |

1. How often do you change your fishing gears? (Every month/season/year/do not change)

______________________________________________________________________

1. Is this your preferred type of gear? Yes No
2. What is your preferred gear type? (dema baskets, spears, nets) ____________________________
3. Have you changed your fishing gear in the last 5 years? Yes No
4. What was the main motivation for changing the gears?________________________________
5. Who introduced this new gear to you? ______________________________________________

### If they own their gear (otherwise go to next sub-section)

1. How much money did you spend on fishing equipment last year? ________________________
2. Total costs with regards to:

Buying the gear _____________________________________________________________

Using this gear on a daily/monthly basis ________________ Repair _____________________

1. Have these costs changed in the last year? (Increased/Decreased/Stayed the same)

________________________________________________________________________

1. On average, how many days did you spend fishing in one month (2012-3)?

Peak season: _______________________ Normal season: ___________________________

Off season: ________________________

1. Do you share your catch with your crew? Yes No

### Hired by someone else

1. What gear you were using before joining this crew? _______________________________
2. How often do you change crews/boats? ________________________________________
3. Did you know head of fishing committee/ captain of the ship or some crew members before joining? ____________________________________________________________________
4. On average, how many days did you spend fishing in one month (2012-3)?

Peak season: _______________________ Normal season: ___________________________

Off season: ________________________

1. What percentage of catch does the crew gets? ____________________________________

### Catch

1. What is your average income per fishing trip? _______________________________________

Peak season: _______________________ Normal season: ___________________________

Off season: ________________________

1. In your view, has your income from fishing increased, remained stable or decreased in the last year? ___________________________________________________________________
2. Average fishing duration per trip

Peak season: _______________________ Normal season: ________________________

Off season: ________________________

1. Did you camp outside your village for fishing in the last year? Yes No
2. How do you rate yourself in the following activities?

| Fishing | Diving/Snorkling | Swimming |
| --- | --- | --- |
|  |  |  |

1= Very good; 2= good; 3= Average (so-so); 4= Poor; 5 = Very Poor

### Knowledge/opinions about gears

1. Which of the following gears have you or any one you knew used in the last 5 years?

|  | Trap Dema | Fixed traps | Spear & Stick | Gill nets | Cast nets | Drag net | Seine nets | Handline | Harpoons | Longlines (Kaputi) |
| --- | --- | --- | --- | --- | --- | --- | --- | --- | --- | --- |
| You |  |  |  |  |  |  |  |  |  |  |
| family |  |  |  |  |  |  |  |  |  |  |
| friends |  |  |  |  |  |  |  |  |  |  |

1=Yes; 0=No

#### Main reasons for changing gears

1. When deciding about fishing gears, how would you rank these different factors? Rank from 1 to 4:

Costs Damage to the sea/fisheries Catch Effort/skills required to operate

# Social and physical capital

1. Number of other session members you know by name (out of 12) __________________ Friends_________________ Family _____________

*Scale:* 1 = strongly agree, 2 = Agree, 3 = Not sure, 4 = Disagree, 5 = strongly disagree

1. In general, I trust people in this village __________________________
2. I usually trust people __________________________

## Physical capital

1. Do you have electricity in your house? Yes No

| TV | VCR/DVD | Refrigerator |
| --- | --- | --- |
| Electric fan | Radio/cassette player | Mobile phone |

Lighting

| Nothing | Kerosene wick | Kerosene lamp | Light bulb |
| --- | --- | --- | --- |

Transport

| Bicycle | Motorcycle | Vehicle | Other: |
| --- | --- | --- | --- |

Roof material

| Thatch | Metal | Tile | Other: |
| --- | --- | --- | --- |

Floor material

| Dirt/soil | Bamboo/palm | Plank Wood | Cement | Finished (tiles, etc.) |
| --- | --- | --- | --- | --- |

1. **Cell phone Questionnaire**
2. Who do you talk to/sms to most through your cell phone? _________________________________
3. How would you characterize your cell phone usage?

Very frequent user frequent user Use sometimes not a lot very rarely

1. How much do you spend on cell phone credit in one day/one week/one month? ________________
2. Do you find cell phone important?

Yes, always Yes, sometimes No, most of the times No, never

1. Cell phone has made life easier? Yes No
2. Do you use ezypaisa/ other services? Yes No
